# Overview of tRNA Modifications in Chloroplasts

**DOI:** 10.3390/microorganisms10020226

**Published:** 2022-01-20

**Authors:** Maxime Fages-Lartaud, Martin Frank Hohmann-Marriott

**Affiliations:** 1 Department of Biotechnology, Norwegian University of Science and Technology, N-7491 Trondheim, Norway; martin.hohmann-marriott@united-scientists.org; 2 United Scientists CORE (Limited), Dunedin 9016, Aotearoa, New Zealand

**Keywords:** chloroplast, genetic code, tRNA modifications, codon, anticodon

## Abstract

The chloroplast is a promising platform for biotechnological innovation due to its compact translation machinery. Nucleotide modifications within a minimal set of tRNAs modulate codon–anticodon interactions that are crucial for translation efficiency. However, a comprehensive assessment of these modifications does not presently exist in chloroplasts. Here, we synthesize all available information concerning tRNA modifications in the chloroplast and assign translation efficiency for each modified anticodon–codon pair. In addition, we perform a bioinformatics analysis that links enzymes to tRNA modifications and aminoacylation in the chloroplast of *Chlamydomonas reinhardtii*. This work provides the first comprehensive analysis of codon and anticodon interactions of chloroplasts and its implication for translation efficiency.

## 1. Introduction

Chloroplasts are a fascinating platform for gaining insights into fundamental aspects of biology as well as for biotechnological applications [1,2,3,4]. The attraction of chloroplasts is based in part on the relative simplicity of the chloroplast genome and protein synthesis machinery. 

**Figure 1:**
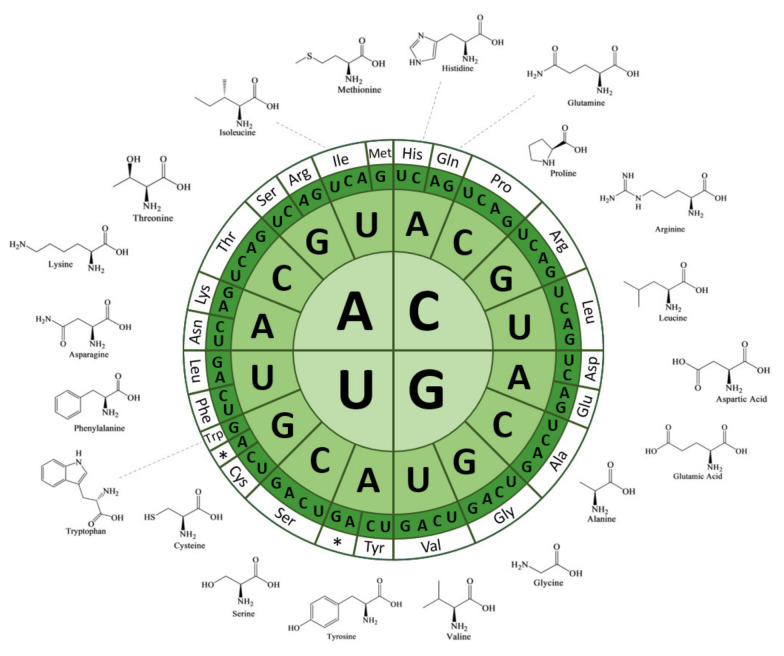
Circular representation of the genetic code. Codons are read from the center letter toward the outer layer. The codons boxes are associated with the amino acids they encode. Amino acid structures are represented next to their respective boxes.

The genetic information contained in DNA and its corresponding mRNA is encoded by sequences of nucleotides (A, T/U, C and G). These nucleotide coding sequences are translated into proteins to fulfill specific molecular functions. The genetic code connects nucleotide bases with a biological signification, i.e., amino acids. The genetic code is organized into nucleotide triplets called “codons” that are used as encryption units for each amino acid. Mathematically, triplets of four nucleotides offer 4^3^ = 64 possible combinations for encryption. However, each codon is not associated with a unique amino acid. In most organisms, 61 codons encode for a total of 20 amino acids and the last three are used as translation termination signals (UAA, UAG and UGA) (Figure 1). As one amino acid is encoded by several codons, a redundancy emerges in the genetic code.

There are two main reasons for this redundancy. The first reason is that 20 amino acids apparently provide sufficient chemical diversity to fulfill protein functional and structural features [5,6]. The second reason is that a triplet-based code not only provides enough encryption capacity, but the excess of combinations is necessary to maintain the fidelity of the genetic code. Indeed, the genetic code is segregated into codon families or “boxes”, due to the absence of strong discrimination by tRNA anticodons. Therefore, most amino acids are associated with several near-cognate codons in duet, triplet or quartet boxes; and some are even decoded by two boxes (Figure 1).

**Figure 2:**
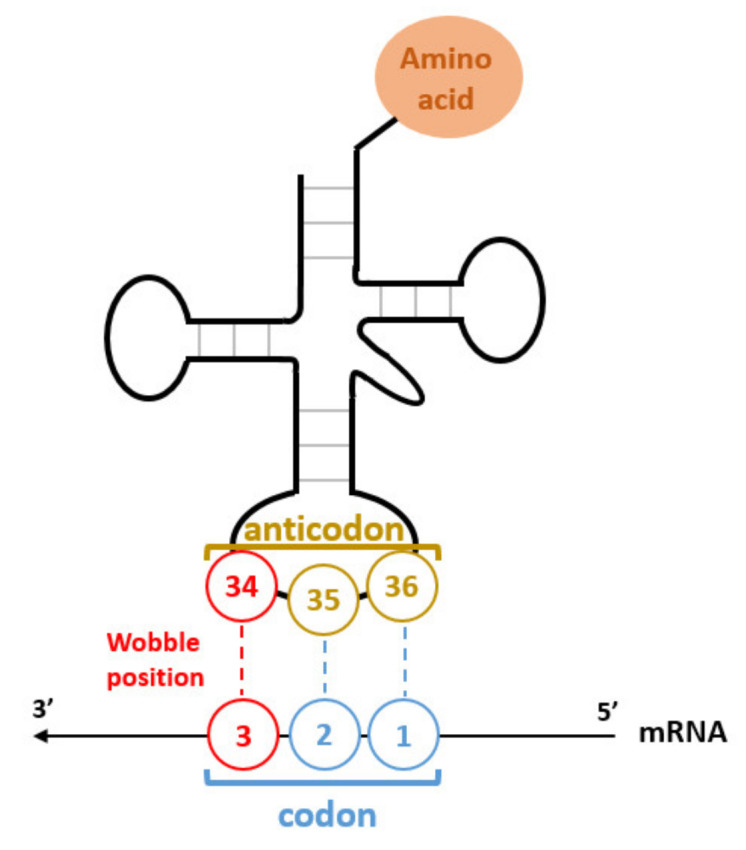
Schematic representation of the codon–anticodon interaction. The first two nucleotides of the codon (N_1_ and N_2_) form a Watson–Crick pair with the anticodon positions N_36_ and N_35_. The interaction in wobble position (N_3_:N_34_) is less stringent, and therefore allows decoding of quartet boxes by superwobbling. The anticodon position 34 is subject to post-transcriptional modification to restrict or expand its decoding properties.

The fidelity of the genetic code is ensured by two mechanisms, codon–anticodon recognition and correct tRNA aminoacylation. For the first mechanism, codons are decrypted through the complementary pairing with a tRNA anticodon. Codon nucleotides in positions 1, 2 and 3 pair with the anticodon positions 36, 35 and 34 respectively (Figure 2). The first and second nucleotide positions of the codon are associated with base 36 and 35 of the anticodon strictly according to the Watson–Crick complementarity rules (A:U, U:A, G:C, C:G) [7]. In contrast, the interaction between the third codon position and the first anticodon base (N_34_) allows a more flexible set of combinations expressed in the wobble rules [7,8,9]. In addition, a wide variety of post-transcriptional nucleotide modifications affects the anticodon loop especially in the wobble position (N_34_) and in the dangling base (N_37_) adjacent to the anticodon [10,11,12,13]. Modifications in the wobble position (N_34_) modulate the pairing properties of the anticodon [11,14,15,16], while those in the dangling position N_37_ provide anticodon stacking and avoid cross talk with symmetric boxes [12,15,17,18]. In 1973, Jukes hypothesized that the genetic code evolved from a primitive form coding for 10 amino acids [17]. Crosstalk between some symmetric boxes was possible due to nucleotide pairing rules, namely G:U/C and U:A/G/U pairing between the first codon base and the third position of the anticodon (N_36_) (Figure 2). It was proposed that some boxes remained unassigned or decoded by lack of specificity or ribosome frameshifts [19,20]. The appearance of new tRNA isoforms along with tRNA modifications permitted to segregate the genetic code into more defined codons boxes (Figure 1). Importantly, tRNA modifications play a fundamental role in the recognition of the appropriate tRNA species during aminoacylation [21,22,23,24] and during translation through structural features of ribosomes that go beyond codon–anticodon recognition [25,26,27,28].

**Figure 3:**
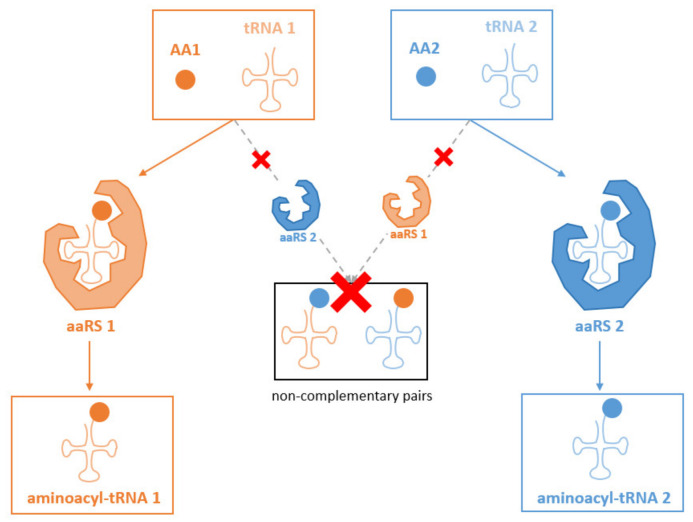
Tripartite complementarity of aaRS. The tripartite complementarity of aaRS enzymes with a specific tRNA-amino acid couple (AA: amino acid, tRNA) is presented. These enzymes do not charge other tRNA species, nor charge other types of amino acids on their cognate tRNA. This specificity is a crucial mechanism maintaining genetic code fidelity.

The second mechanism that safeguards the fidelity of the genetic code is the correct association of a tRNA with its corresponding amino acid. Aminoacylation of tRNAs is a highly specific reaction that is accomplished by aminoacyl tRNA synthetases (aaRSs). Each tRNA aminoacylation is performed by a corresponding aaRS that recognizes specifically one tRNA species and ligates the corresponding amino acid [29,30] (Figure 3). There are two classes of aaRS (class I and II) that vary widely in sequence and structural features, pointing toward distinct phylogenic origins [31,32,33]. Each class of aaRSs recognizes opposite sides of the tRNA acceptor stem (minor or major groove) [34]. The two classes are subdivided into three subclasses (a, b and c) based on mechanistic properties, anticodon-binding domain features and conserved structural motifs [35]. Each class of aaRS is responsible for the aminoacylation of half of the 20 proteogenic amino acids. Aminoacylation is a two-step mechanism in which an aaRS catalyzes the ATP-dependent activation of a specific amino acid, forming an aminoacyl-adenylate intermediate, that is subsequently ligated onto the 3’ end of the corresponding tRNA [36,37]. The structural features leading to the activation of amino acids are conserved within each aaRS class [38]. However, the recognition mechanisms of each amino acid differ substantially among aaRSs [29]. The binding site of an aaRS is usually composed of two sub-domains, each involved in one respective step of tRNA aminoacylation [29]. The enzymatic specificity of the aminoacylation process is a major determinant of the emergence of the genetic code and is an essential step within protein biosynthesis [39], because aminoacyl-tRNAs must consistently carry the same amino acid. Non-covalent interactions play a crucial role in the specific recognition of the correct amino acid and the tRNA species [40,41,42]. It is important to note that the recognition of tRNAs by aaRS enzymes is sensitive to nucleotide modifications occurring on the tRNA, particularly on the anticodon [21,22,23,24]. Interestingly, aaRS enzymes are also involved in non-canonical activities such as cell signaling, RNA splicing, translational and transcriptional regulation.

Chloroplasts originate from the endocytosis of an ancestral prokaryote, that is affiliated with modern cyanobacteria, by a eukaryotic cell [43,44,45]. The eukaryotic host enslaves chloroplasts as power plants by transferring essential prokaryotic genes to the nucleus and rerouting gene products to the chloroplast [1,46]. Therefore, the genome of the chloroplast (plastome) is considerably reduced [47,48], limiting its genetic information to proteins that require co-translational assembly into a multi-protein complex or the association with cofactors such as chlorophyll [49]. Despite the genome reduction, chloroplasts sustain their own translation machinery, which is able to decipher the genetic code with a minimal set of 24 tRNA species by superwobbling [50,51]. The plastidial tRNA set follows the evolutionary strategy that consists in a total depletion of tRNA harboring A_34_ and C_34_ in the anticodon [16]. This minimalist strategy conserved a single tRNA species containing G_34_ and U_34_ in anticodons to decrypt duet and quartet codon boxes [16].

Microorganisms with minimal genomes (<1 Mb) often utilize a reduced tRNA set for protein translation. The same evolutionary pressure appears to be experienced by organelles, such as the chloroplasts or mitochondria, which maintain a small genome (200 kb and 20 kb resp.) and a simple translation machinery that allows for complete decoding of the genetic code (with some exceptions for mitochondria). *Mycoplasma capricolum* possesses a minimal genome (1 Mb) that shows striking resemblance to the plastome based on their similar tRNA set characteristics and AT-content equilibria [13,15,52]. *E. coli,* on the other hand, possesses a more complex genome with 43 different tRNA species, of which many have several genomic copies. Decryption the *E. coli* genome relies primarily on tRNA isoacceptor concentrations and to a lower extend on codon–anticodon affinity [53]. By contrast, minimal genomes often possess single tRNA copies and mainly rely on codon–anticodon affinity to decipher the genetic code. In the latter case, the characteristics of the anticodon loop, especially nucleotide modifications at base N_34_ and N_37_, ensure accurate mRNA decoding and influence codon usage [12,13,14,15,16,52]. The study of the translation machinery of minimal organisms provides fundamental insights into codon–anticodon recognition and protein synthesis dynamics. Furthermore, this knowledge is necessary for synthetic biology approaches, such as heterologous protein expression and genome recoding schemes. We currently do not have a comprehensive overview of tRNA modifications and the enzymes involved in tRNA maturation (aminoacylation and modifications) for chloroplasts. Here we focus on establishing the tRNA modifications of the green algae *Chlamydomonas reinhardtii.* This choice is based on the wide breath of available data that is present for this organism and its promise as a platform for biotechnological applications.

As only few tRNA modifications have been conclusively demonstrated in *Chlamydomonas reinhardtii*, we assembled a detailed picture of tRNA structural modification using molecular data from published studies of the chloroplast of algae and plants. We correlated this information with bioinformatics analyses concerning the availability of genes that mediate required biochemical reactions and their prospective targeting to the chloroplast of *Chlamydomonas reinhardtii*. We cross-validated the information gathered on tRNA modifications with codon–anticodon translation efficiency to obtain a coherent picture of the deciphering of the genetic code in algal chloroplasts.

## 2. Materials and Methods

The tRNA modifications present in chloroplasts were investigated by performing a meticulous literature review of demonstrated tRNA modifications from biochemical analytical data in various chloroplasts. In order to cross-validate these data or find other potential tRNA modifications, we searched for enzymes implicated in tRNA modifications and tRNA charging. *C. reinhardtii* nuclear genes coding for aaRS, release factors and tRNA modification enzymes that may be directed to the chloroplast were identified from JGI online resource Phytozome (phytozome.jgi.doe.gov/ (accessed on 5 December 2021)). The analytical data found in the literature, describing plastidial tRNA modifications, and enzymes identified as responsible for these transformations were used to suggest biochemical modifications. In order to discriminate the proteins targeted to the chloroplast from the proteins that remain in the eukaryotic context, we performed chloroplast-targeting analysis (https://services.healthtech.dtu.dk/service.php?TargetP-2.0 (accessed on 10 December 2021)). Based on the scores from TargetP, we estimated the potential presence of these enzymes in the chloroplast (Supplemental Datasheet). Protein blast (blast.ncbi.nlm.nih.gov/Blast.cgi (accessed on 5 December 2021)) was used to obtain *C. reinhardtii* enzymes that are homologous to identified chloroplastic enzymes in other species (such as *Arabidopsis thaliana*, *cyanobacteria* and other algal species). Chloroplastic aaRS genes were previously identified in *A. thaliana* [54], hence, these enzymes were compared to *C. reinhardtii* aaRS enzymes by protein blast. The highest percentage of identity permitted to establish with confidence which enzymes were cytosolic or targeted to the chloroplast *C. reinhardtii* (Supplemental Datasheet). The sequence of tRNA genes found in the chloroplast are presented in Supplemental Table S1. Secondary tRNA structures and potential uridine modifications were predicted with tRNAmod (see Supplemental Figure S1) (webs.iiitd.edu.in/raghava/trnamod/index.html (accessed on 10 December 2021)).

Relative translation efficiency of each codon within each codon box was inferred from literature describing codon–anticodon energy stability or codon decoding rates from ribosome profiling studies (referred in the main text), and/or derived from codon usage evolution (only for quartet boxes possessing modified or unmodified U_34_) from chloroplast and *Mycoplasma* [16,52].

## 3. Results and Discussion

A significant amount of tRNA bases are post-transcriptionally modified, allowing complex interactions that go beyond the standard base-pairing rules. Modifications occur within the anticodon loop as well as on the rest of the tRNA. All these modifications play crucial roles in the recognition of tRNA by aaRS enzymes and by the ribosome.

### 3.1. Common Nucleotide Modifications of tRNA Backbones in Chloroplasts

Common modifications of chloroplast tRNA that have been observed outside the anticodon arm include 2-o-oxymethyl-Guanine Gm_18_, pseudouridine ψ_55_, ψ_26_ and ψ_27_, methyl guanidine m^7^G_46_, m^2^G_10_, dihydrouridine D_(16-20-21-47)_ [55,56,57,58,59,60,61]. The stem preceding the anticodon loop is very often composed of a Watson–Crick base pair at position referred as B_29-41_, followed by a G/C Watson–Crick pair at B_30-40_ to provide a stable helical conformation. One base pair further, either a C=G or a A–ψ pair is preferred at B_31-B39_ [15]. At the start of the anticodon loop, B_32_ and B_38_ form a non-Watson–Crick pair. The former being a pyrimidine (U, C) occasionally modified to ψ, Cm or Um; and the latter being most frequently an A or less often a C or ψ [62]. Notably, the invariant residue U_33_ constitute the U-turn between the 5′ and 3′ helical stacks of the anticodon loop [12]. These characteristics are widely found among bacteria and thought to maintain the biophysical properties of the stem preceding the anticodon loop [15]. A list of all plastidial tRNAs and their predicted structures is presented in Supplemental Table S1 and Figure S1. We investigated the nuclear genome of *C. reinhardtii* to identify genes involved in the various tRNA modifications. Each enzyme was subject to a subcellular targeting analysis to evaluate their potential presence in the chloroplast (Supplemental Datasheet).

### 3.2. Modifications of the Anticodon-Adjacent Nucleotide (N_37_) Maintains the Fidelity of the Genetic Code

Modifications of the “dangling” base in position 37 affects the stability and specificity of the anticodon loop. Its purpose is to increase stacking of the anticodon first nucleotide in order to ensure proper pairing of N_1_:N_36_ [10,63]. These modifications are usually dependent on the first base of the codon (N_1_). In the case of C_1_NN codons, with the exception of arginine codons, the purine 37 of each tRNA is a guanine that is invariably changed to N1-methyl-guanosine (m^1^G_37_) in all three kingdoms of life [15]. Hence, in the chloroplast of *C. reinhardtii*, tRNAs of proline (UGG), leucine (UAG), histidine (GUG) and glutamine (UUG) all possess m^1^G_37_ [57,59,61,64,65], while arginine (ICG) contains m^6^A_37_ [65,66]. The 1-methylguanosine in position 37 (m^1^G_37_) is determinant for the quality of codon–anticodon pairing as well as the avoidance of frameshifts for C-starting codons (C_1_NN) [12,67].

All other codons are decrypted with tRNAs containing an adenine in position 37. In the chloroplast of *C. reinhardtii*, A_37_ can be modified to 2-methylthio-N6-isopentenyladenosine (ms^2^i^6^A_37_), N6-threonylcarbamoyladenosine (t^6^A_37_) or N6-methyladenosine (m^6^A_37_) [16,55,56,57,58,59,60,64,65,67,68,69,70]. In many organisms, it is generally modified when the adjacent base B_36_ is either an A or a U; this pattern of modification correlates with the necessity to stabilize the weak neighboring codon–anticodon pair A_1_:U_36_ and U_1_:A_36_ [10,12,15,63]. 

For the first modification, MiaA and MiaB are the enzymes known to modify A_37_ to i^6^A_37_ and ms^2^i^6^A_37_, respectively, for all U-starting codons (U_1_NN) in bacteria [71,72,73], namely Tyr, Phe, Cys, Ser (UCN), Trp and Leu (UUG/A). The role of this modification is to maintain correct U_1_:A_36_ pairing and prevent any symmetric crosstalk [12,17]. We found one orthologue of MiaA and two of MiaB that have moderate to high likeliness to be translocated to the chloroplast (Supplemental Datasheet). The i^6^ or ms^2^i^6^A_37_ modification were confirmed experimentally for tRNA-Phe [59], tRNA-Trp [58], tRNA-Tyr [68] and tRNA-Cys [69] in plant and algal chloroplasts.

Two additional adenine modifications t^6^A_37_ and m^6^A_37_ have been identified on chloroplastic tRNAs but their respective enzymes, which modify base 37, remain unknown is *C. reinhardtii*. A potential candidate for this function is the gene Cre10.g455400, which is annotated as coding for an enzyme adding m^6^A to tRNA-Val (however, valine was not shown to contain m^6^A_37_, but the enzyme may act on other tRNAs) and displays features that may allow it to be translocated to the chloroplast (Supplemental Datasheet). The residue t^6^A_37_ plays a role for preventing G_1_:U_36_ mispairing for all A_1_NN codons therefore ensuring fidelity of the genetic code. m^6^A_37_ has a similar role but applies to various tRNAs [12,17]. In chloroplasts, the t^6^A_37_ was identified in tRNA-Ile (GAU) [60] and (k^2^C_34_AU) [56], tRNA-Lys [55], tRNA-Thr_3_ is modified in spinach to m^6^t^6^A_37_ [70] while the tRNAs for Asn, Ser (AGU/C) and Arg (AGA/G) have not been investigated. Finally, m^6^A_37_ occurs in tRNA-Met_e_ (in line with experimental data for tRNA-Arg-ICG) [55,66] while A_37_ of tRNA-Met_i_ remains unmodified [74].

In contrast to the aforementioned modifications, plastidial tRNAs reading G_1_NN codons do not possess modifications of A_37_ [55,75,76], with the exception of tRNA-Asp [77] which has not been identified. Indeed, according to nucleotide pairing rules [7], cytosine strictly pairs with guanosine, which is sufficient to prevent frameshifts and mispairing of N_1_:C_36_.

The modifications affecting the dangling base are crucial to maintain genetic code accuracy. However, modifications occurring in the anticodon, especially in base 34, are of particular importance because they dictate tRNA decoding properties. These modifications result either in a higher codon–anticodon specificity or a less stringent recognition for superwobbling [10].

### 3.3. Nucleotide Modifications Affecting Anticodons Shape Their Deciphering Properties

In the next section we analyze all plastidial tRNA species with their anticodon modifications regarding their effect on decoding properties.

#### 3.3.1. The Anticodon Base 34 of Single Codon Box

Amino acids encoded by a single codon require a high codon–anticodon specificity to avoid misreading of near-cognate codons. Only methionine (AUG) and tryptophan (UGG) possess a single codon. For both amino acids, the anticodon of the plastidial tRNA ends with C_34_, which, according to the wobble theory [7], reads only G-ending codons.

In the case of methionine, there are actually two distinct tRNAs, one optimized for translation initiation (tRNA-Met_i_) [78] and one for polypeptide elongation (tRNA-Met_e_). The former contains a ψ in position 39 and an unmodified anticodon loop [74], while in the latter, ψ is found in position 32 and the aforementioned m^6^A_37_ residue [55,66]. Interestingly, in some prokaryotes C_34_ can be modified to ac^4^C_34_ to maintain accurate translation of only the AUG codon [79,80], although near-cognate codon reading is rare. While an orthologue of an RNA cytidine acetyltransferase is present in the nuclear genome (Cre03.g192850) the modification was not shown to appear on tRNA-Met [66] but was detected in rice chloroplast lysate [81]. Hence, C_34_ certainly provides enough discrimination to read only the AUG codon.

The tRNA of tryptophan contains ψ in position 39 and 38, an unmodified C_34_CA anticodon and an i^6^A_37_ or ms^2^i^6^A_37_ modification [58]. In eubacteria, C_34_ is usually modified to Cm_34_ to avoid misreading of UGA stop codons [16]. The absence of UGA codons in the chloroplast genome may also indicate that tRNA-Trp could interact slightly with this codon without detrimental consequences. For example, in *M. capricolum*, the two codons UGG and UGA encode tryptophan while RF2 has been lost, showing the lack of requirement for efficient discrimination between the two codons [16,82]. We hypothesize that the enzyme performing the modification to Cm_34_/Um_34_ is Cre10.g417650, which is likely shuttled to the chloroplast (Supplemental Datasheet); however, the enzymatic product was found only on tRNA_Leu_-Um_34_AA. Either the modification was not detected by Canaday et al. at the time [58] or it is not present and the low affinity A_1_:G_34_ does not interfere with RF2 termination as demonstrated by Young et al. [83] if RF2 is present at all in chloroplasts.

The last case to discuss in this section is the isoleucine tRNA reading AUA codons. Isoleucine is encoded by three codons, AUU/C and AUA, which are recognized by two distinct tRNA species. The tRNA reading AUA actually originates from a tRNA with methionine-like anticodon C_34_AU. It is post-transcriptionally modified at position 34 into k^2^C_34_ (lysidine) [21,56,84]. Thus, tRNA-Ile-(k^2^C_34_AU) accomplishes specific decoding of AUA codons for isoleucine. The enzyme responsible for lysidination was identified as Cre13.g572800 and is predicted to be exported to the chloroplast (Supplemental Datasheet).

#### 3.3.2. The Anticodon Base 34 of NNU/C Duet Codon Boxes Are Mostly Unmodified

Duet codon boxes are divided into two groups, pyrimidine-ending codons (NNU/C) and purine-ending codons (NNA/G). In the chloroplast, within each (NNU/C) box, codons are decoded with a single tRNA containing G_34_ in the first position of the anticodon. This group is composed of the amino acid boxes coding for Phe, Cys, Tyr, Asp, His, Asn, Ile (AUC/U) and Ser (AGC/U). All these codons possess less than two G or C in codon positions N_35_ and N_36_, which infers a low to intermediate codon–anticodon binding energy [15]. The anticodon G_34_NN recognizes NNC_3_ codons by Watson–Crick pairing and less efficiently NNU_3_ codons by U_3_:G_34_ wobble pairing [15,85,86,87,88]. The C_3_:G_34_ Watson–Crick pair forms three hydrogen bonds, compared to two for the U_3_:G_34_ wobble pair, which increases the codon–anticodon binding energy and its stability. This increase in codon–anticodon energy and the Watson–Crick geometry improves codon recognition by the ribosome, thus allowing faster translation of the C_3_:G_34_ Watson–Crick by the ribosome [28,88].

Interestingly, there is evidence pointing to the presence of queuine (Q) instead of G_34_ in certain plant tissues or under particular growth conditions [77,89,90]. To corroborate these observations, two queuine tRNA-ribosyltransferases were identified in the nuclear genome of *C. reinhardtii* and one of them (Cre12.g558400) showed a high probability of being transferred to the chloroplast. This enzyme acts on all tRNAs harboring a U_35_ in the second anticodon position (His, Asn, Asp and Tyr). Nonetheless, queuine has not been found in the tRNA of His and Asn and is only present in specific conditions for Asp and Tyr in plants [77,89,90]. One explanation for this is that most eukaryotes, including *C. reinhardtii*, are not able to synthesize queuine. Instead, eukaryotes rely on an external bacterial source for queuine supply. Interestingly, a salvage pathway was identified in *C. reinhardtii* [91]. In queuine replete conditions, *C. reinhardtii* may incorporate Q_34_ in place of G_34_ in G_34_U_35_N plastidial tRNAs. This may underlay specific gene expression regulation due to the ability of queuine (Q_34_) for decoding NNC/U codons that differs from G_34_ [92]. Indeed, G_34_ pairs preferentially with NNC compared to NNU [15,85], while Q_34_ exhibits less bias between C_3_ and U_3_ deciphering depending on the context of the anticodon loop [8,86]. Interestingly, tRNA modifications play a role in differential gene expression in response to a stress factor [93,94].

#### 3.3.3. The Anticodon Base 34 of NNA/G Duet Codon Boxes Restricts Deciphering to Purine Codons

In the second group of duet boxes, NNA/G codons are decoded by single tRNA containing a modified U_34_. This group is composed of the 2-codon boxes of Lys, Glu, Gln, Arg (AGA/G) and Leu (UUA/G). In bacteria, the most common modifications of U_34_ are 5-iminomethyl-U_34_ derivatives (nm^5^U, mnm^5^U, cmnm^5^U). In addition, the same uridine is sometimes thiolated (s^2^U). We identified candidate enzymes in *C. reinhardtii* responsible for the addition of cmnm^5^, mnm^5^s^2^ and cmnm^5^s^2^ to U_34_ that are likely to be shuttled to the chloroplast (Supplemental Datasheet). These modifications affect the tRNAs of Glu, Gln, Lys and probably Arg (AGA/G) [55,64,75]. Although the thiolation of U_34_ generally affects the tRNAs of glutamate, lysine and glutamine in bacteria [95], U_34_ thiolation was previously detected solely for glutamate in chloroplasts [75]; however, the inaccuracy of the chromatographic method used for the lysine and glutamine plastidial tRNAs [55,64] may leave some place for the presence of thiolation on U_34_ of these two tRNAs. The bacterial tRNA-Leu (UUA/G) also contains the cmnm^5^s^2^ group on U_34_ [15], although it was not found in plastid versions [61]. However, just as its bacterial counterpart, the plastidial tRNA-Leu (UUA/G) contains a 2′-O-methyluridine (Um_34_) in the wobble position [61]. These different U_34_ modifications restrict the reading to purine codons with a strong preference for A-ending codons [8,15,16,96,97,98,99]. 

#### 3.3.4. The Anticodon Base 34 of Quartet Codon Boxes Expands tRNA Reading Properties by Superwobbling

In the chloroplast of *C. reinhardtii*, each amino acid box encoded by four codons is read by a single respective tRNA. Only the glycine box possesses an additional tRNA (anticodon GCC) that was shown to be dispensable [51]. Decoding of four-codon boxes requires the presence of at least one G or C in position 35/36 of the anticodon [12,100] and other important residues in the anticodon loop such as C_32_ [101,102]. These residues increase the pairing strength between codon and anticodon, making the third codon base less significant for decoding [15]. According to the superwobble theory, a single tRNA containing U_34_ is able to read all codons of quartet family boxes [12,13,50,51,100]. In a majority of bacteria, U_34_ is modified to xo^5^U derivatives [15,16]. In *E. coli*, it is modified to cmo^5^U_34_ that provides the ability to read all four codons of the family boxes [103,104,105]. However, synthesis of these xo^5^U derivatives were not found in *C. reinhardtii* by mass spectrometry [81]. Remarkably, *Mycoplasma* and mitochondria use unmodified U_34_ to read the quartet boxes [15]. The chloroplast shows striking resemblance to the *M. capricolum* tRNA set and the reading properties within quartet boxes due to its minimalistic features and similar GC content [15]. In *M. capricolum*, modifications such as s^2^U_34_ and Um_34_ are always absent from this type of tRNA to prevent steric hindrance, thus making these tRNA less stringent at the wobble position [16,105]. In the chloroplast, U_34_ is modified in the tRNAs of Pro, Ala, Val and potentially Ser (UCN_3_) and Thr as well, but the precise nature of the modification remains unidentified [57,65,76]. The tRNA of glycine (U_34_CC) contains an unmodified U_34_ able to read all four codons with a reasonable efficiency, although less efficient decoding of some codons may be the reason why the second tRNA (G_34_CC) is maintained in the plastome [55]. Additionally, it was demonstrated that the presence of C_32_ is crucial in helping tRNA-Gly to decode all four codons [101,102]. In the case of leucine (CUN_3_), U_34_ is not modified; however, G_36_ is methylated to m^7^G_36_ [59,61]. This modification allows tRNA-Leu (UAm^7^G_36_) to read the four codons of the associated box. Additionally, base 37 is modified to m^1^G_37_ to provide 5′ stacking power to the anticodon [59].

The above-mentioned tRNA species containing an unmodified U_34_ nucleotide efficiently recognize A-ending codons, whilst the G_3_:U_34_ wobble base is fairly unstable [15], C-ending codons present a poor pairing efficiency [106], and there is a lack of data for U-ending codons. The deciphering properties of the other tRNAs from quartet boxes, harboring the unknown modification of U_34_, have not been investigated. However, the deciphering properties can be deduced from the codon usage of quartet boxes containing a unique deciphering tRNA. Indeed, according to Ikemura, the evolutionary forces shaping codon usage are tRNA concentrations, in the case of several tRNA isoacceptors for the same amino acid; or codon–anticodon affinity in the case of a single tRNA decoding an entire codon box [53,107]. Here, the tRNA set of quartet boxes of the chloroplast follows the second case: the higher the codon–anticodon affinity, the higher the codon usage. The codon usage of the chloroplast of *C. reinhardtii* is strongly biased toward A- and U-ending codons for quartet boxes [52], as for *M. capricolum* [12,15]. Even though evolutionary mutational bias leads to a higher AT composition in the chloroplast, there is an active pressure toward a strong enrichment of A- and U-ending codons for quartet boxes [52]. Therefore, we hypothesize that anticodons containing modified or unmodified U_34_ lead to higher translation efficiency of A- and U-ending codons within quartet boxes in the chloroplast of *C. reinhardtii* [52].

At last, we investigate the special case of the arginine box (CGN_3_). Here, the first base of the anticodon is modified from uridine to inosine (I_34_) [55,65,108]. This gives the tRNA the ability to decode preferentially U-ending codons; inosine also pairs well with CGC codons, but poorly recognizes CGA. It has been shown that I_34_ can decode NNG_3_ albeit very inefficiently [66,104]. This might explain the extremely low occurrence of CGG codon in the plastome (only three occurrences). Other chloroplast-containing organisms such a liverwort retained a tRNA-Arg (C_34_CG) that can read the CGG codon more efficiently [109].

**Figure 4:**
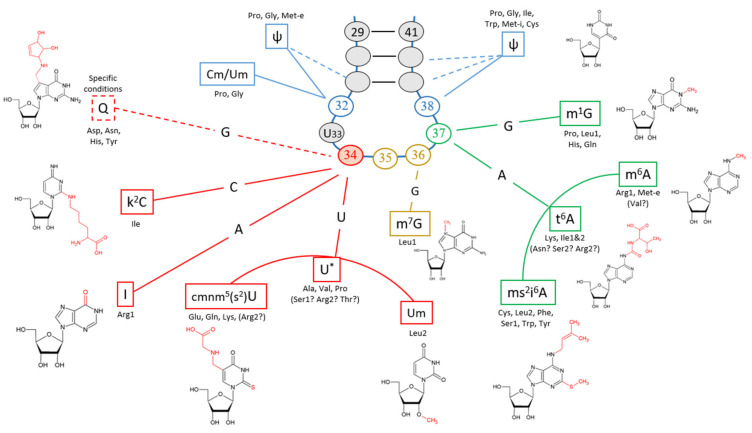
Modifications of the tRNA anticodon loop. The most important modifications of the tRNA anticodon loop are presented in relation to their positions. Each position is associated with corresponding modifications depending on the original nucleotide that was present before modification. The amino acids affected are indicated below each box (when several tRNA are present for one amino acids: 1 refers to the quartet box and 2 refers to the duet box; for isoleucine 1 represent the duet box and 2 the AUA codon). Modifications occurring in the stem at bases 31–32 and 38–40 are indicated in blue. All tRNAs contain the invariable U_33_. Modifications at position 37 are represented in green; t^6^A_37_, m^6^ A_37_ and ms^2^i^6^ A_37_ for adenine [16,55,56,57,58,59,60,64,65,67,68,69] and m^1^G_37_ for guanosine [57,59,61,64,65]. The last two positions of the anticodon (36 and 35) are represented in gold, modified only for leucine CUN at position 36 into m^7^G_36_ [59,61]. The wobble base, which is the target of the most important modifications, is represented in red. Each type of nucleotide at position 34 of the anticodon is associated with its corresponding modifications and the affected aminoacyl-tRNA. Namely, Um_34_, cmnm^5^U_34_/cmnm^5^s^2^U_34_ [55,64,75] and U*_34_ (* represents an unidentified modification) [57,65,76] for uridine; Inosine (I) replacing the adenosine 34 in the arginine tRNA-I_34_CG [55,65,108]; Cytosine modification into k_2_C_34_ for the isoleucine tRNA-k_2_C_34_AU [56]. Queuosine is represented in dashed lines because of the lack of solid evidence [77,89,90], thus remaining hypothetical. In theory, this modification affects tRNA of the type G_34_U_35_N (His, Asn, Asp and Tyr).

**Figure 5:**
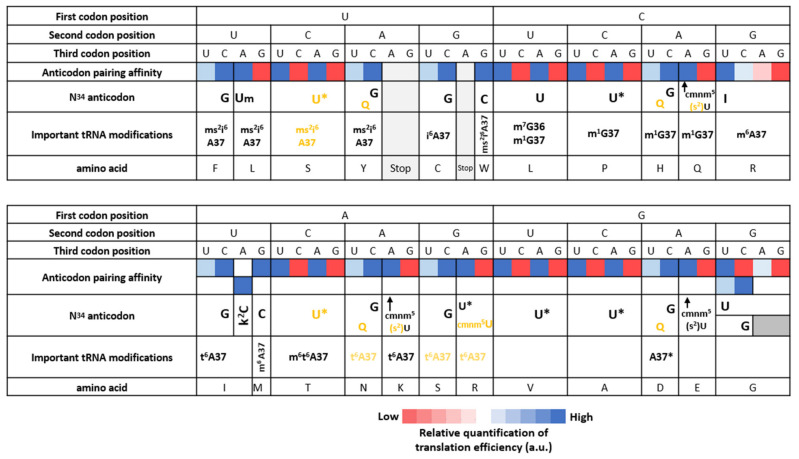
Codon–anticodon pairing efficiency based on energy stability. The relative translation efficiency of each codon within each codon box is inferred from literature (see main text) and/or derived from codon usage evolution (quartet boxes) from chloroplast and *Mycoplasma*. The tRNA modifications affecting anticodon position 34 and other important modifications (presented in Figure 4) are detailed in separate rows of the table. Base 34 of each anticodon is represented directly below the codon it recognizes by Watson–Crick pairing. The modifications determined experimentally are shown in black and hypothesized modifications suggested by our analysis are shown in yellow. Asterisk next to a nucleotide refers to an unknown modification.

We summarized the most important modifications of the anticodon loop in Figure 4. Additionally, we present the deciphering properties of each tRNA based on the translation efficiency of their respective codons (Figure 5).

### 3.4. Translation Termination

In prokaryotes and organelles, there are two distinct release factors responsible for terminating translation. The first release factor (prfA/RF1) recognizes UAA and UAG codons while the second (prfB/RF2) recognizes UAA and UGA and halt peptide synthesis [110,111]. Interestingly, the specificity of the release factors RF1 and RF2 toward stop codons is ensured by the tripeptides Pro-Ala-Thr and Ser-Pro-Phe respectively [112]. Release factors possess a universally conserved Gly–Gly–Gln (GGQ) motif that is responsible for the hydrolysis of the ester bond between the last tRNA in the ribosomal P-site and the peptide [113]. Among the 67 coding sequences present in the chloroplast, only three genes (*psbL*, *clpP*, *ftsH*) possess a UAG stop codon while all the other genes use UAA to stop translation. It is interesting to note that the Opal stop codon UGA is not present at all. This led to the hypothesis that only release factor 1 may perform translation termination [83]. Both release factors prfA (Cre16.g673617 or Cre06.g289350) and prfB (Cre01.g010864) are nuclear-encoded. While it remains unclear if the *prfA* gene product is effectively translocated to the chloroplast, protein alignments show that Cre16.g673617 is more closely related to RF1 from *Synechocystis PCC 6803* and to AtcpRF1 from *Arabidopsis thaliana,* the latter of which has been shown to be translocated into the chloroplast [114] (Supplemental Data S3). Surprisingly, *C. reinhardtii* retained *prfB* (Cre01.g010864), which bioinformatics analysis indicates is likely being targeted to the chloroplast (Supplemental Data S3) and may still provide active plastidial UGA termination. It was demonstrated that UGA occurrence increases compared to UAG relatively to the abundance of RF2, suggesting that in this case RF2 concentration is low or inexistent [115]. In addition, organisms with very high AT3 content (i.e., third codon position), like the chloroplast, tend to evolve toward decreasing RF2 concentration close to zero and axiomatically drive the reassignment of UGA codons to UAA that is consistently over-represented in highly expressed genes [115]. Young et al. showed that UGA opal codons can be translated as tryptophan when a genomically integrated tRNA-_Trp-UCA_ containing a modified UCA anticodon is expressed [116]. A temperature-sensitive tRNA was used to engineer a cold-inducible translational system (CITRIC) responding to the reassigned UGA codons [116]. These special characteristics regarding translation termination constitute an opportunity for stop codon reassignment, where the newly freed codon is used to integrate non-canonical amino acids into proteins.

### 3.5. Comparison of tRNA Modifications within Minimal Genomes

Here, we investigate the nucleotide modification strategies of the chloroplast and other minimal genomic systems (mitochondria and *M. capricolum)* as well as the eubacterial model *E. coli* (Table 1). All these systems possess the m^1^G_37_ modification for the C_1_NN codons. When adenine is present in position 37, the chloroplast follows similar strategies as the bacteria and mitochondria with slight exceptions. For U_1_NN codons, the chloroplast utilizes the modifications ms^2^i^6^A_37_ and i^6^A_37_ that are also present in mitochondria and *E.coli*. For C_1_NN and G_1_NN codons, the chloroplast presents similar tRNA characteristics as *Mycoplasma* with m^6^A_37_ and unmodified A_37_. Some differences occur for A_1_NN codons, where the chloroplast displays the m^6^A_37_, unmodified A_37_ as in *Mycoplasma*. Furthermore, the chloroplast possesses a t^6^A_37_ modification instead of ct^6^A_37_ found in the three other organisms, which have similar functional implications. The chloroplast also contains the m^6^t^6^A_37_ modification that is present only in *E. coli*. Mitochondria display the lowest amount of modifications in base 37, while the chloroplast possesses similar characteristics as *M. capricolum*, at the notable exceptions of m^6^t^6^A_37_ and ms^2^i^6^A_37_ that resemble the more complex organism *E. coli*.

The plastidial modifications occurring on base N_34_ are similar to the ones occurring in *M. capricolum*, with a few interesting differences (Table 1). Within the NNU/C duet boxes, the two bacteria and the two organelles use unmodified G_34_ to read both codons, except for codons containing A_35_ (NA_35_U/C). For these codons, tRNAs from *E. coli* and mitochondria harbor the queuosine modification that might be present in chloroplast under specific growth conditions. The NNA/G duet boxes are decrypted using the restrictive groups cmnm^5^, cmnm^5^s^2^ added to U_34_ or Um_34_, which are similar deciphering strategies as *E. coli* and *Mycoplasma*. Interestingly, mitochondria opted for different modifications, τm^5^ and τm^5^s^2^, which have the same restrictive deciphering properties as in plastids. When it comes to quartet boxes, *E. coli* utilizes the cmo^5^ modification of U_34_ to read all four codons of the quartet box but also contains additional tRNAs harboring unmodified C_34_ and G_34_. In contrast, organelles and *Mycoplasma* decipher the quartet boxes with tRNAs containing unmodified U_34_, known to confer superwobbling [50,51], which is a common strategy for decoding minimal genomes. Strikingly, the chloroplast displays an unknown modification of U_34_, different from cmo^5^, that can decipher all codons of quartet boxes. In the case of methionine, the chloroplast and *M. capricolum* display an unmodified cytosine to read uniquely the AUG codon, which may offer enough discrimination through Watson–Crick base pairing to avoid reading near-cognate codons; *E. coli* evolved the ac^4^C_34_ modification for more accurate codon discrimination, while the mitochondrial modification f^5^C_34_ reads both AUG and AUA. The last-mentioned AUA codon is allocated to isoleucine using the k_2_C_34_ modification for the three other examples. Last, in the case of tryptophan, the chloroplast adopts a strategy consisting of an unmodified C_34_, while *E. coli* possesses the Cm_34_ modification, both sufficient to read only the UGG codon. *M. capricolum* and mitochondria possess the cmnm^5^Um_34_ and τm^5^U_34_ modifications respectively, which also assign the UGA codon to tryptophan instead of stop signal.

Overall, the chloroplast shares several tRNA modifications with other minimal genomic systems.

## 4. Conclusions

This study presents a comprehensive view of codon–anticodon interactions in the chloroplast of *C. reinhardtii*. Many of the experimentally established and postulated tRNA deciphering strategies and their basis in tRNA modifications for *C. reinhardtii*, are likely also found in the chloroplasts of other members of the Viridiplantae. The nature of these tRNA modifications defines how the genetic code is deciphered in chloroplasts. Each modification presents specific aptitudes in reading a codon family based on codon–anticodon pairing affinity and interactions with ribosomes. The chloroplast displays a complex interplay of evolutionary forces that lead to a minimum set of tRNAs, which is modified to maintain tRNA-aminoacyl synthesis fidelity, as well as tRNA–ribosome recognition and protein synthesis.

The high codon–anticodon affinity is associated with a decreased codon decoding time during translation [15,117], thus improving protein yield. The relationship between codon–anticodon pairing affinity and protein yield has been recognized in the codon usage of highly expressed genes [52]. Appreciating and manipulating this interplay is vital for realizing the potential of heterologous gene expression and biotechnological applications in the chloroplast.

## Figures and Tables

**Table 1:** Comparison of the tRNA modifications occurring in base N_34_ and N_37_ for chloroplast, mitochondria, *M. capricolum* and *E. coli*. Compiled information collected in this paper for the chloroplast and sourced from Grosjean et al. for the other organisms [15]. The type of codons affected by the modifications is indicated on the lefthand side of the table. Asterisk next to a nucleotide refers to an unknown modification.

Modified Base		Codons		*E. Coli*	*M. Capricolum*	Mammalian Mitochondria	Chloroplast
N37	A37	(U1NN)		ms^2^i^6^ A		ms^2^i^6^ A	ms^2^i^6^ A
					m^6^ A	i^6^ A	i^6^ A
						unmodified A	
		(C1NN)		m^2^ A	m^6^ A	unmodified A	m^6^ A
		(G1NN)		m^2^ A	m^6^ A		A*
				unmodified A	unmodified A	unmodified A	unmodified A
		(A1NN)		m^6^t^6^ A			m^6^t^6^ A
				ct^6^A	ct^6^A	ct^6^A	t^6^A
					m^6^ A		m^6^ A
					unmodified A	unmodified A	(unmodified A)
	G37	(C1NN)		m^1^G	m^1^G	m^1^G	m^1^G
N34		NNU/C (duet boxes)	NNU/C	unmodified G	unmodified G	unmodified G	unmodified G
			NAU/C	Q	unmodified G	Q	unmodified G (maybe Q)
				GluQ			
		NNA/G (duet boxes)		mnm^5^s^2^U			
				mnm^5^Um	cmnm^5^U	τm^5^U	cmnm^5^U
				cmnm^5^s^2^U	cmnm^5^s^2^U	τm^5^s^2^U	cmnm^5^s^2^U
				cmnm^5^Um	cmnm^5^Um		Um
				Cm			
		NNU/A/C/G (quartet boxes)		cmo^5^U	unmodified U	unmodified U	unmodified U
				unmodified G			U*
				unmodified C			
		quartet Arginine		I	I	unmodified U	I
		NNG	Trp	Cm	Cm	τm^5^U	unmodified C
					cmnm^5^Um		
			Met	ac^4^C	unmodified C	f^5^C	unmodified C
		Isoleucine AUC		k_2_C	k_2_C	(NA)	k_2_C

## Supplementary Materials

The following supporting information can be downloaded at: www.mdpi.com/article/10.3390/microorganisms10020226/s1, Figure S1: Secondary structure and potential modifications of tRNAs from the chloroplast of *Chlamydomonas reinhardtii*; Table S1: List of tRNAs found in the chloroplast of *Chlamydomonas reinhardtii*; Supplemental Data contains the following sections: tRNA list, tRNA copy number, aaRS list and targeting, release factors, tRNA modification enzymes.
