# Supplementary material for: Overview of tRNA Modifications in Chloroplasts
Source: Microorganisms. 2022 Jan 20;10(2):226. doi: 10.3390/microorganisms10020226 (PMC8877259; doi:10.3390/microorganisms10020226)
Supplement: Supplementary file 1 [file microorganisms-10-00226-s001.zip › tRNA supplementals/Supplemental Figures modifications.pdf]

Supplemental Tables and Figures

Overview of tRNA modifications in chloroplasts

Fages-Lartaud et al. 2022

Supplemental Table S1: List of tRNAs found in the chloroplast of *Chlamydomonas reinhardtii*……… *p2*

Supplemental Figure S1: Secondary structure and potential modifications of tRNAs from the chloroplast of *Chlamydomonas reinhardtii*……………………………………………………………………………….. *p3-5*

Supplemental Table S1: List of tRNAs found in the chloroplast of *Chlamydomonas reinhardtii.* (source: http://plantrna.ibmp.cnrs.fr/plantrna)

tRNA-Ala (GCA)	GGGGATATAGCTCAGTTGGTAGAGCGCTGCCTTTGCAAGGCAGATGTCAGCGGTTCGAATCCGCTTATCTCCACCA
tRNA-Arg (AGA)	GGGTTCATCGTTCAATGGATAGGACAGCGGCCTTCTAAGCCGTCAATGTAGGTTCGATTCCTACTGGGCCTGCCA
tRNA-Arg (CGT)	GAGCTTGTAGCTCAGTGGACTAGAGCACATGGCTACGAACCATGGGGTCGGGGGTTCGAAACCCTCCTGGCTCGCCA
tRNA-Asn (AAC)	TCTTCAGTAGCTCAGTGGTAGAGCGATCGGCTGTTAACCGATTGGTCGTAGGTTCAAGTCCTACCTGGGAGTCCA
tRNA-Asp (GAC)	GGGATTGTAGTTCAATTGGTTAGAGCACCGCCCTGTCACGGCGGAAGTTGCGGGTTCGAGTCCCGTCAATCCCGCCA
tRNA-Cys (TGC)	GGCGGCATAGCAAGCGGTAAGGCCGTGGATTGCAAATCTCTATTCCCCAGTTCAAATCTGGGTGCCGCCTCCA
tRNA-Gln (CAA)	TGGGGCGTCGCCAAGTGGTAAGGCTGCGGTTTTTGGTACCGCCATTCGCAGGTTCGAATCCTTCCGCCCCAGCCA
tRNA-Glu (GAA)	GCCCCCATCGTCTAGAGGCCTAGGACACCTCCCTTTCACGGAGAAAACGCGGATTCGAATTCCGCTGGGGGTACCA
tRNA-Gly (GGC)	GCGGACATAGCTCAATGGTAGAGTATTTCCTTGCCAAGGAAAATGTTGCGGGTTCGACTCCCGTTGTCCGCTCCA
tRNA-Gly (GGA)	GCGGATGTAACTCAATCGGTAGAGTGCGATCCTTCCAAGTTCGAGGTTGTGGGTTCGAGTCCCATCATCCGCTCCA
tRNA-His (CAC)	GGCGGGCGTAGCCAAGTGGTAAGGCAGTGGATTGTGACTCCACTATTCGCGGGTTCGAACCCCGTCGTTCGCCCCA
tRNA-Ile (ATA)	GCACTGTTGGCCGAGCGGATGAGGCAAACGACTCATAATCGTTATAAGGTAGGTTCAACTCCTATACGGTGCACCA
tRNA-Ile (ATC)	GGGCTATTAGCTCAGTTGGTTAGAGCGTTGCTTTGATAAGGCAAAAGTCGAAAGTTCAAATCTTTCATAGCCCACCA
tRNA-Leu (TTA)	GGGGATATGGCGGAATGGTAGACGCTACGGACTTAAAATCCGTTCTTGTGCGAACAAGGTGAGGGTTCAAGTCCCTC TTTCCCCACCA
tRNA-Leu (CTA)	GCCTTCGTGATGGAACTGGTAGACATCCTGGTTTTAGGAACCAGTGCTGAAAGGCGTGCCGGTTCAAATCCGGCCGAA GGCACCA
tRNA-Lys (TTT)	GGGTTGCTAACTCAATGGTAGAGTACTCGGCTTTTAACCGATAAGTTCTGGGTTCGAGTCCCAGGTAACCCACCA
tRNA-Met-e (ATG)	GCCTGCTTAGCTCAGTTGGTTAGAGCGTCCGTTTCATAAGCTGATTGTCACTAGTTCAAATCTAGTAGCAGGCACCA
tRNA-Met-i (ATG)	AGCAGGGTAGAGCAGTCTGGTAGCTCGTGGGGCTCATAATCCTGAGGTCGCAGGTTCAAATCCTGCCCCTGCCACCA
tRNA-Phe (TTC)	GCCGGGATAGCTCAGTTGGTAGAGCAAAGCGTTGAAAATGCTTGGGTCACCGGTTCAAGTCCGGTTCCTGGCACCA
tRNA-Pro (CCA)	CGGGATGTAGCGCCAGCTTGGTAGCGCATGTGCTTTGGGAGCATAGGGTCGCAGGTTCGAATCCTGTCATCCCGACCA
tRNA-Ser (TCA)	GGAAAGGTGGCAGAGTGGTTAATTGCACCAATTTTGAAAATTGGCGTGGCTTCGCGGTCACCGAGGGTTCGAATCCCT CCCTTTCCGCCA
tRNA-Ser (AGC)	GGAGAGATGGCTGAGTGGTCGAAAGCGGCTGATTGCTAATCAGTTTAAGTGCGACTTGCGCTTACGAGGGTTCGAAT CCCTCTCTCTCCGCCA
tRNA-Thr (ACA)	GCTTGCTTAACTCAATCGGTAGAGTATCGGTTTTGTAAACCGAAGGTTATCGGTTCAACTCCGATAGCAAGCTCCA
tRNA-Trp (TGG)	ACGTCCTTAGTTCAGTCGGTAGAACGCAGGTTTCCAAAACCTGATGTCGTGGGTTCAATTCCTACAGGGCGTGCCA
tRNA-Tyr (TAC)	GGGTCGATGCCCGAGTGGTTAATGGGGGCGGATTGTAAATCCGTTGACTTAGTCTGCGTTGGTTCGAATCCGACTCGA CCCACCA
tRNA-Val (GTA)	AGGCCCATAACTCAGTCGGTAGAGTGATTGCCTTACAAGCAATAGGTCATCGGTTCAAGTCCGGTTGGGCCTACCA

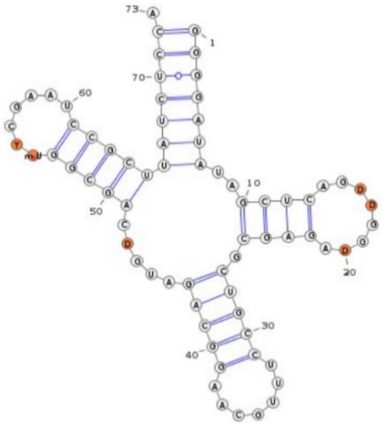

1.Ala_TGC

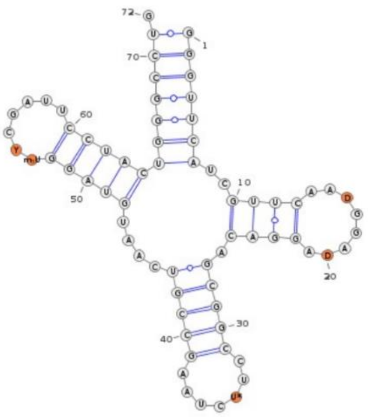

2.Arg_TCT

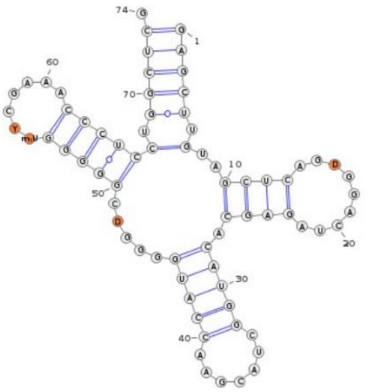

3.Arg_ACG

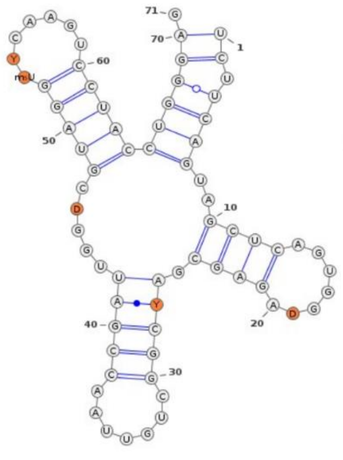

4.Asn_GTT

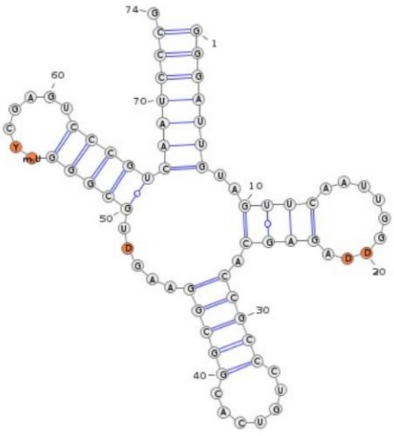

5.Asp_GTC

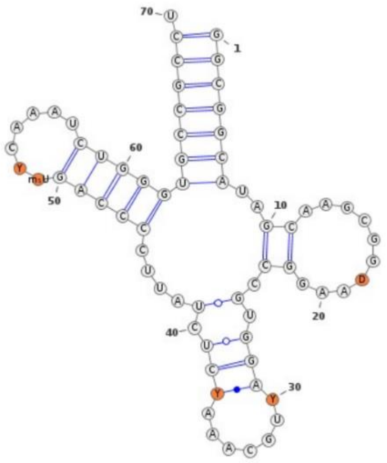

6.Cys_GCA

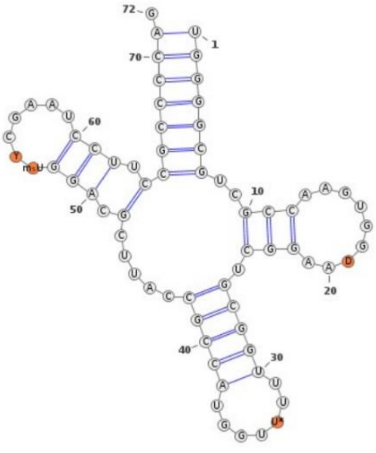

7.Gln_TTG

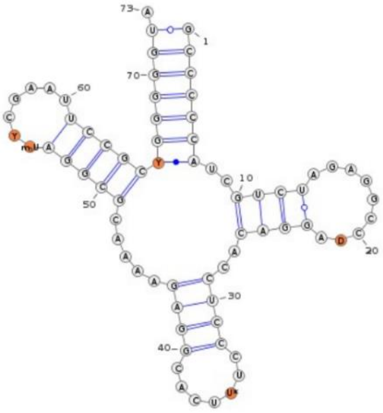

8.Glu_TTC

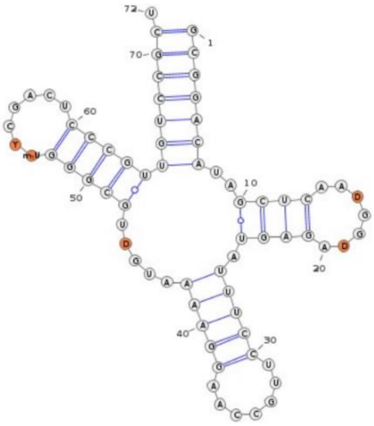

9.Gly_GCC

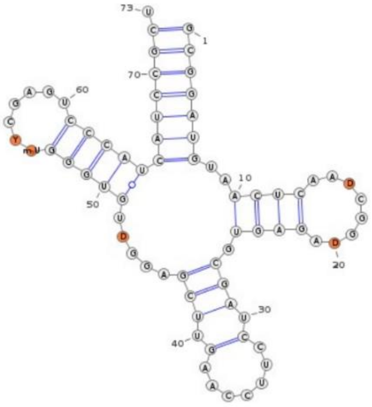

10.Gly_TCC

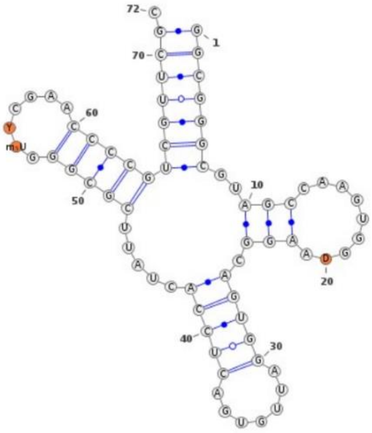

11.His_GTG

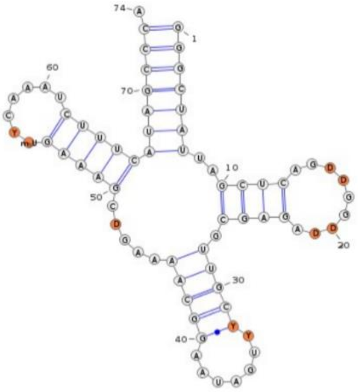

13.Ile_GAT

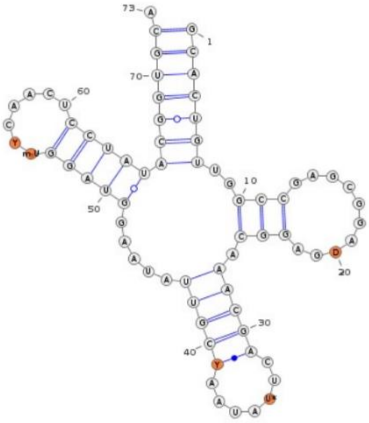

1.Ile_TAT

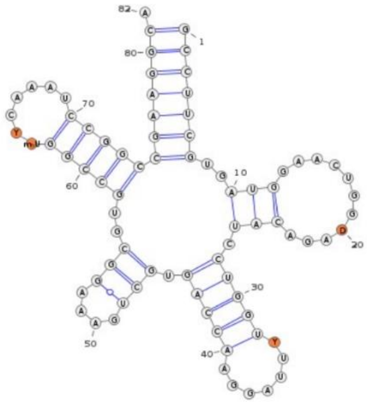

15.Leu_TAG

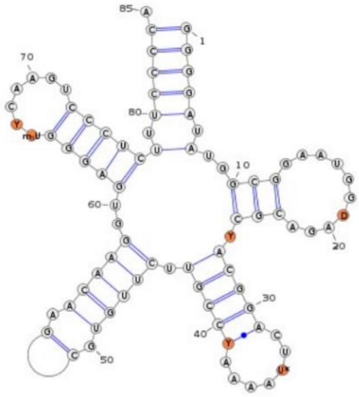

14.Leu_TAA

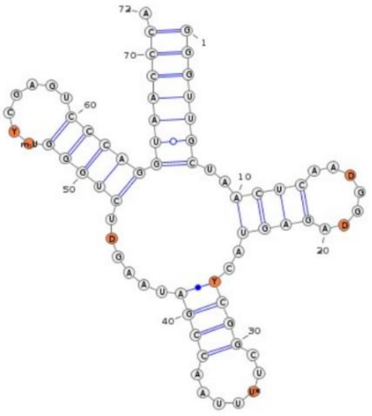

16.Lys_TTT

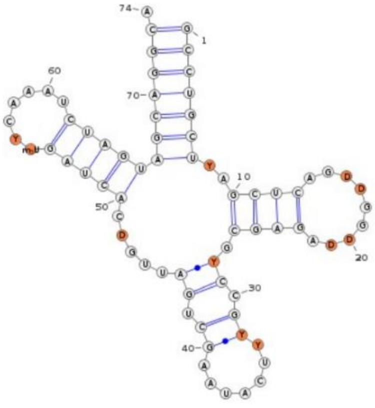

17.Met_CAT_e

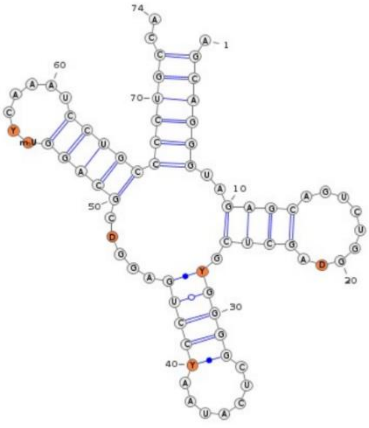

18.Met_CAT_i

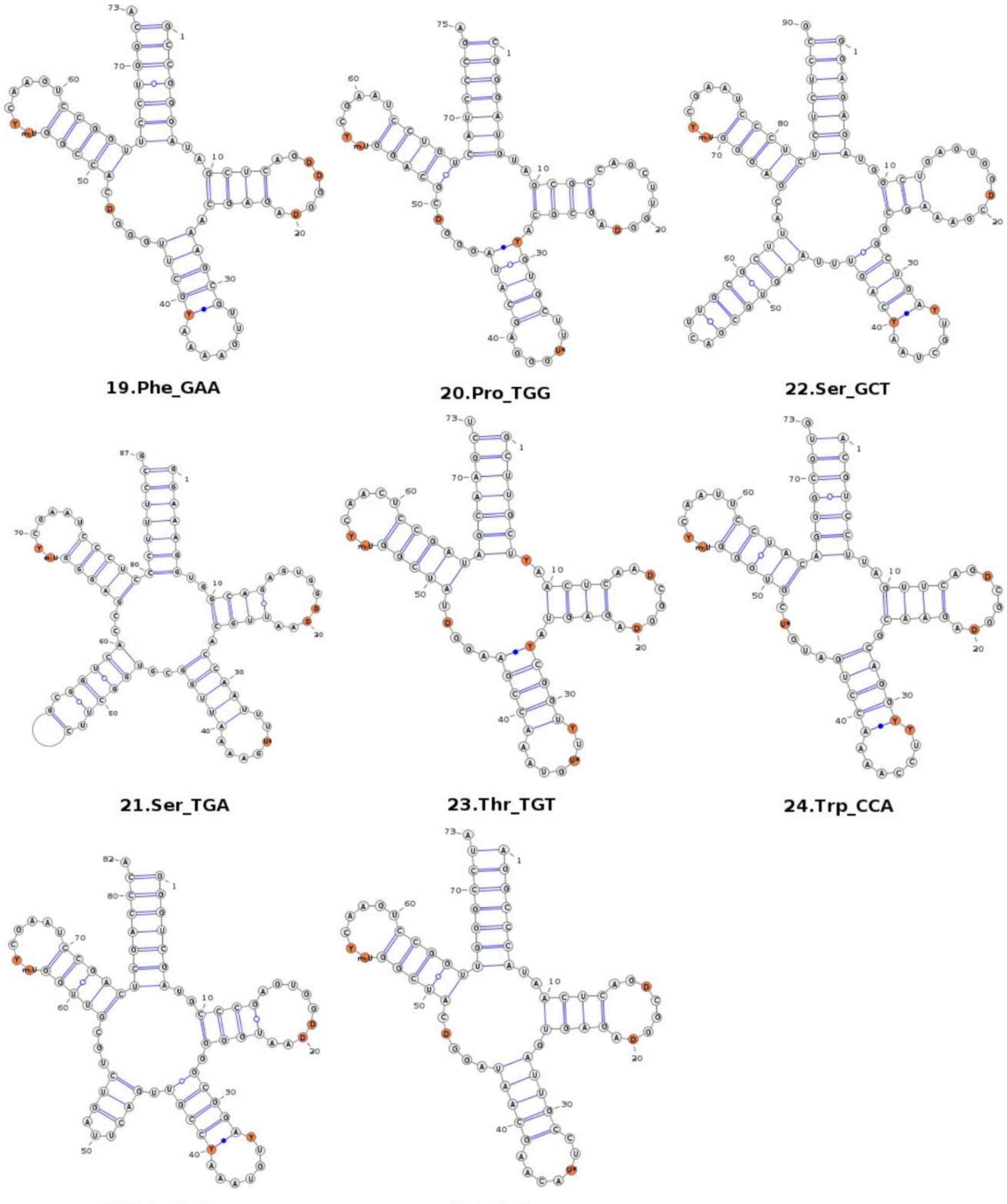

Supplemental Figure S1: Secondary structure and potential modifications of tRNAs from the chloroplast of *Chlamydomonas reinhardtii*. The anticodon of the tRNAs appears next to the encoded amino acid below each structures. Modifications are highlighted in orange and changed to their potential new nucleoside. The outcome of modified bases might contain errors due to the computation program used.

Computed with tRNAmod (https://webs.iiitd.edu.in/raghava/trnamod/index.html).
